# Persistent Arthralgia Associated with Chikungunya Virus Outbreak, US Virgin Islands, December 2014–February 2016

**DOI:** 10.3201/eid2304.161562

**Published:** 2017-04

**Authors:** Leora R. Feldstein, Ali Rowhani-Rahbar, J. Erin Staples, Marcia R. Weaver, M. Elizabeth Halloran, Esther M. Ellis

**Affiliations:** Fred Hutchinson Cancer Research Center, Seattle, Washington, USA (L.R. Feldstein, M.E. Halloran);; University of Washington, Seattle (A. Rowhani-Rahbar, M.R. Weaver, M.E. Halloran);; Centers for Disease Control and Prevention, Fort Collins, Colorado, USA (J.E. Staples);; US Virgin Islands Department of Health, Saint Croix, US Virgin Islands (E.M. Ellis)

**Keywords:** chikungunya virus, chikungunya fever, vector-borne infections, persistent arthralgia, alphavirus, outbreak, viruses, US Virgin Islands, comparative study, long-term sequelae

## Abstract

After the 2014–2015 outbreak of chikungunya virus in the US Virgin Islands, we compared the prevalence of persistent arthralgia among case-patients and controls. Prevalence was higher in case-patients than controls 6 and 12 months after disease onset. Continued vaccine research to prevent acute illness and long-term sequelae is essential.

Autochthonous transmission of chikungunya virus (CHIKV) was first reported in the Americas in December 2013 on the island of Saint Martin, and by early 2016, the virus had spread to 45 countries in the Caribbean and Central, South, and North America (*1*,*2*). Acute symptoms of CHIKV infection often resolve within 7–10 days (*3*). However, 7%–79% of case-patients from previous outbreaks have reported persistent arthralgia for months after infection (*4*–*8*). Persistent arthralgia associated with CHIKV illness has been assessed in persons in Southeast Asia, South America, and Europe but not in the Caribbean (*4*,*9*).

The US Virgin Islands (USVI), one of many regions in the Caribbean affected by the CHIKV epidemic, identified its first locally acquired case in June 2014 (*10*). USVI subsequently reported nearly 2,000 suspected CHIKV cases before the last laboratory-confirmed case was reported in February 2015 (*11*). To determine the prevalence of CHIKV-associated persistent arthralgia in USVI, we compared the prevalence of persistent arthralgia among CHIKV case-patients and nonsymptomatic controls during December 2014–February 2016.

## The Study

A confirmed case-patient was defined as a USVI resident of any age with acute onset of fever (>38°C) and severe arthralgia or arthritis not explained by another medical condition plus 1 of the following: 1) CHIKV RNA in blood, as determined using reverse transcription PCR, or 2) CHIKV-specific IgM antibodies in serum, as determined using ELISA in conjunction with either CHIKV-specific neutralizing antibodies using plaque reduction neutralization test with a 90% cutoff or CHIKV-specific IgG using ELISA (*10*). Confirmed case-patients captured by the USVI Department of Health surveillance system were invited via telephone to participate in a follow-up investigation at 6 and 12 months after acute illness; a total of 165 case-patients were recruited. Verbal informed consent was obtained at the start of each interview.

At the 12-month follow-up, we concurrently enrolled a nonsymptomatic control group. Members of the control group were recruited from the emergency department waiting room of a hospital or from a health clinic in USVI; the group comprised 167 USVI residents of any age. Persons were not eligible for the control group if they reported symptoms consistent with CHIKV disease (i.e., concurrent fever and acute joint pain) or responded “yes” to being tested for CHIKV and test results were positive. All controls were offered free CHIKV IgG testing and were excluded from analysis if positive (Technical Appendix Table 1).

At 6 and 12 months, we interviewed case-patients about presence, frequency, and anatomic location of arthralgia after acute CHIKV infection. We defined persistent arthralgia as joint pain occurring at least once per week within 1 month before the interview. We asked case-patients whether they had a history of arthritis (defined as doctor-diagnosed arthritis before CHIKV illness). At the 12-month interview, we asked case-patients whether they had difficulty performing everyday activities (i.e., walking, climbing stairs, lifting heavy objects, getting in and out of cars, opening jars). We interviewed the control group only once, using the same 12-month questionnaire that we used for case-patients. The University of the Virgin Islands and the University of Washington ethics committees approved this study.

At each time point, we ran 2 independent regression models. We constructed generalized linear models, using the binomial family with robust variance estimators, to estimate prevalence differences and prevalence ratios. We included age grouping (<35, 36–55, and >55 years), sex, and self-reported history of arthritis in the models. We used 2-sample *t*-tests with unequal variances to determine differences in arthralgia prevalence by affected joint sites. We used Stata 14.0 (StataCorp LLC, College Station, TX, USA) for the analysis.

**Figure 1:**
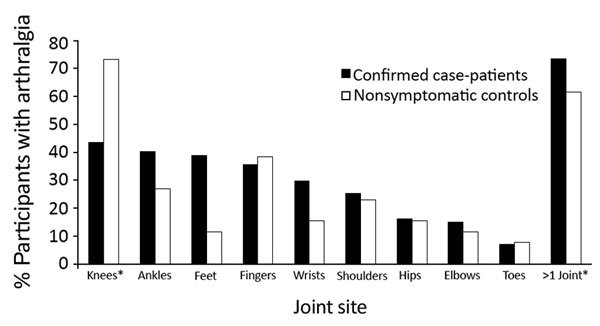
Arthralgia reported by joint site among confirmed chikungunya virus case-patients 6 months after illness onset and by nonsymptomatic controls enrolled at the time of the 12-month follow-up for case-patients, US Virgin Islands, December 2014–February 2016. *Statistically significant differences (p<0.01) between case-patients and controls.

**Figure 2:**
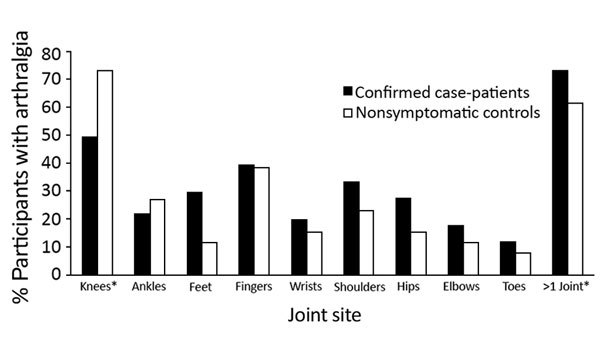
Arthralgia reported by joint site among confirmed chikungunya virus case-patients 12 months after illness onset and by nonsymptomatic controls enrolled at the time of the 12-month follow-up for case-patients, US Virgin Islands, December 2014–February 2016. No statistically significant differences were found between case-patients and controls.

Six months after disease onset, the difference in persistent arthralgia prevalence between case-patients and controls was 32% (95% CI 24%–40%), after adjusting for age, sex, and self-reported history of arthritis; 12 months after onset, the difference was 19% (95% CI 11%–28%) (Table 1). At 6 months, case-patients reported higher prevalence of foot-specific joint pain (28%, 95% CI 11%–44%), and at 12 months, controls reported higher prevalence of knee-specific pain (29%, 95% CI 8%–50%) (Figure 1). At 12 months, we found no statistically significant differences by joint site between case-patients and controls (Figure 2).

**Table 1:** Prevalence and prevalence ratios of persistent arthralgia and impaired physical functionality among residents in a study of persistent arthralgia after a chikungunya virus outbreak, US Virgin Islands, December 2014–February 2016*

Variable	Prevalence difference (95% CI)	p value	Prevalence ratio (95% CI)	p value
Persistent arthralgia				
6 month analysis*	0.32 (0.24–0.40)	<0.001	2.90 (1.90–4.43)	<0.001
12 month analysis*	0.19 (0.11–0.28)	0.001	2.51 (1.71–3.69)	<0.001
Difficulty performing daily activities				
Walking	0.11 (0.03–0.18)	0.007	1.77 (1.06–2.95)	0.028
Climbing stairs	0.12 (0.05–0.19)	0.001	1.81 (1.15–2.86)	0.011
Lifting heavy objects	0.04 (−0.02–0.11)	0.209	1.68 (0.96–2.97)	0.072
Getting in and out of cars	0.09 (0.03–0.14)	0.001	2.65 (1.32–5.32)	0.006
Opening jars	0.15 (0.07–0.23)	<0.001	2.24 (1.28–3.91)	0.005

*Adjusted for age group, sex, and self-reported history of arthritis.

After we adjusted for age, sex, and self-reported history of arthritis, case-patients were 1.77 (95% CI 1.06–2.95) times more likely than controls to have difficulty walking and 1.81 (95% CI 1.15–2.86) times more likely to have difficulty climbing stairs (Table 1). Case-patients were also 2.65 (95% CI 1.32–5.32) times more likely to have difficulty getting in and out of cars and 2.24 (95% CI 1.28–3.91) times more likely to have difficulty opening jars.

## Conclusions

Although acute symptoms of CHIKV infection are well-documented (*12*), information on the prevalence of long-term sequelae remains limited to observational studies, many of which lack comparator groups (*4*). From our year-long study of 165 case-patients and 167 controls, we found an almost 3-fold increased risk for persistent arthralgia in CHIKV case-patients at 6 and 12 months after illness onset. At 12 months, case-patients were significantly (p<0.01) more likely than controls to report difficulty performing daily activities. Our unadjusted persistent arthralgia estimates at 6 (44%) and 12 (33%) months (Table 2) fall within the pooled estimate from a metaanalysis (40%, 95% CI 31%–49%) (*4*).

**Table 2:** Demographic and physical characteristics among residents in a study of persistent arthralgia after a chikungunya virus outbreak, US Virgin Islands, December 2014–February 2016*

Variable	No. (%) chikungunya virus case-patients		No. (%) nonsymptomatic controls at 12-month case-patient follow-up, N = 167*
	6 months after symptom onset, N = 165	12 months after symptom onset, N = 128	
Median age (range), y	52 (1-96)	52 (1-92)	35 (2-78)
Sex			
F	108 (65)	82 (64)	108 (65)
M	57 (35)	46 (36)	59 (35)
Employed or a student	96 (58)	73 (57)	125 (75)
History of self-reported arthritis	37 (22)	30 (23)	28 (17)
Annual household income <US$50,000	–	82 (64)	121 (72)
Joint pain day of interview	59 (36)	34 (27)	14 (8)
Joint pain within month of interview	87 (53)	51 (40)	26 (16)
Difficulty performing daily activities			
Walking	–	36 (28)	20 (12)
Climbing stairs	–	40 (31)	20 (12)
Lifting heavy objects	–	27 (21)	16 (10)
Getting in and out of cars	–	27 (21)	10 (6)
Opening jars	–	33 (26)	13 (8)
Sleeping	27 (31)	19 (37)	8 (29)
Health somewhat/much worse after 1 y	–	28 (22)	16 (10)
Subsample of participants reporting joint pain within mo of interview	N = 87	N = 51	N = 26
Persistent arthralgia	72 (44)	42 (33)	20 (12)
Joint pain frequency			
Daily	42 (48)	28 (55)	13 (50)
2–3 times/wk	17 (20)	8 (16)	5 (19)
1 time/wk	13 (15)	6 (12)	2 (8)
<1 time/wk	12 (14)	9 (18)	6 (23)
Not known	3 (3)	0	0
Symmetrical joint pain	27 (31)	14 (27)	7 (27)
Joint pain time of day			
Morning	16 (18)	12 (24)	2 (8)
Day	10 (11)	5 (10)	1 (4)
Night	11 (13)	6 (12)	5 (19)
Morning and night	5 (6)	2 (4)	1 (4)
Present at all times or activity dependent	43 (49)	23 (45)	15 (58)
Not known	2 (2)	3 (6)	2 (8)
Worst time of day for joint pain			
Morning	29 (33)	21 (41)	3 (12)
Day	9 (10)	7 (14)	1 (4)
Night	20 (23)	8 (16)	8 (31)
Morning and night	3 (3)	3 (6)	0
Present at all times or activity dependent	23 (26)	8 (16)	12 (46)
Not known	3 (3)	4 (8)	2 (8)

*Values are no. (%) except as indicated. –,data not obtained.

Consistent with findings in previous studies, case-patients in our study reported a higher prevalence of foot-specific joint pain, (*6*,*8*,*13*,*14*). Furthermore, a higher proportion of case-patients reported the presence of persistent arthralgia and of more severe arthralgia in the morning; this finding is consistent with those from previous studies reporting a high prevalence of morning stiffness among case-patients (*6*,*8*).

Our comparative study had limitations, which may have influenced our findings. First, due to missing or incorrect contact information (Technical Appendix Table 2), the study sample represents only 36% of eligible confirmed CHIKV case-patients and thus might not be representative of all USVI residents with CHIKV disease. Second, controls were younger than case-patients and were interviewed only once. Thus, we may have overestimated persistent arthralgia among case-patients due to progression of osteoarthritis associated with increased age and may have underestimated persistent arthralgia among controls. However, a BRFSS (Behavioral Risk Factor Surveillance System) survey indicated that 15% (95% CI 14%–17%) of adult USVI residents reported having arthritis (*15*), a percentage consistent with our estimated persistent arthralgia prevalence among controls (17%). Third, we did not test all controls for CHIKV IgG; thus, some controls with asymptomatic CHIKV infection may have been included in the analysis. However, prevalence differences were similar when we included controls who were asymptomatic but IgG-positive (n = 12). Last, most case-patients knew their diagnosis, which may have influenced their reporting of persistence arthralgia, and case-patients with persistent arthralgia may have been more inclined to participate in the interviews. As a result of these limitations, we may have overestimated the association between persistent arthralgia and CHIKV disease up to 1 year after illness onset.

Our results emphasize that, in the USVI, CHIKV illness was associated with persistent arthralgia and difficulty with daily activities 1 year after disease onset. These findings highlight the need for therapeutic and vaccine research to manage and prevent acute illness and long-term sequelae associated with CHIKV infection. The results also underscore the need for studies to identify risk factors for long-term sequelae of CHIKV illness, to estimate the burden of persistent arthralgia after acute illness, and to understand the effect of persistent arthralgia on quality of life.

Technical Appendix Summary of the enrollment process for persons in a study of persistent arthralgia after a chikungunya virus outbreak, US Virgin Islands, December 2014–February 2016.
