# Supplementary material for: Persistent Arthralgia Associated with Chikungunya Virus Outbreak, US Virgin Islands, December 2014–February 2016
Source: Emerg Infect Dis. 2017 Apr;23(4):673–6. doi: 10.3201/eid2304.161562 (PMC5367425; doi:10.3201/eid2304.161562)
Supplement: Technical Appendix — Summary of the enrollment process for persons in a study of persistent arthralgia after a chikungunya virus outbreak, US Virgin Islands, December 2014–February 2016. [file 16-1562-Techapp-s1.pdf]

Article DOI: http://dx.doi.org/10.3201/eid2304.161562

Persistent Arthralgia Associated with Chikungunya Virus Outbreak, US Virgin Islands, December 2014–February 2016

Technical Appendix

Technical Appendix Table 1. Number of nonsymptomatic controls tested for CHIKV antibodies and interviewed

Variable	Nonsymptomatic control group
No. tested for IgG	134
No. IgG negative	33
No. IgG positive*	12
Total interviewed	167

*Persons who tested positive for CHIKV IgG were excluded from the analysis.

Technical Appendix Table 2. Enrollment and follow-up of CHIKV cases from December 2014–February 2016

Enrollment period	No. enrolled
6-mo enrollment	
Laboratory-confirmed CHIKV cases	457
Missing phone number	116
Incorrect phone number or not in service	93
Did not answer the phone after 3 attempts	70
Refused to participate	11
Died	2
Total enrolled in the 6 mo follow-up	165
12-mo follow-up	
CHIK cases interviewed at 6 mo	165
Incorrect phone number or not in service	9
Did not answer the phone after 3 attempts	24
Refused to participate	4
Total enrolled in the 12 mo follow-up	128
